# Dual targeting of microtubule and topoisomerase II by α-carboline derivative YCH337 for tumor proliferation and growth inhibition

**DOI:** 10.18632/oncotarget.3264

**Published:** 2015-03-27

**Authors:** Jun-Mei Yi, Xiao-Fei Zhang, Xia-Juan Huan, Shan-Shan Song, Wei Wang, Qian-Ting Tian, Yi-Ming Sun, Yi Chen, Jian Ding, Ying-Qing Wang, Chun-Hao Yang, Ze-Hong Miao

**Affiliations:** ^1^ Division of Antitumor Pharmacology, State Key Laboratory of Drug Research, Shanghai Institute of *Materia Medica*, Chinese Academy of Sciences, Shanghai 201203, People's Republic of China; ^2^ Division of Medicinal Chemistry, State Key Laboratory of Drug Research, Shanghai Institute of *Materia Medica*, Chinese Academy of Sciences, Shanghai 201203, People's Republic of China

**Keywords:** YCH337, α-carboline derivative, microtubule, topoisomerase II, antitumor activity

## Abstract

Both microtubule and topoisomerase II (Top2) are important anticancer targets and their respective inhibitors are widely used in combination for cancer therapy. However, some combinations could be mutually antagonistic and drug resistance further limits their therapeutic efficacy. Here we report YCH337, a novel α-carboline derivative that targets both microtubule and Top2, eliciting tumor proliferation and growth inhibition and overcoming drug resistance. YCH337 inhibited microtubule polymerization by binding to the colchicine site and subsequently led to mitotic arrest. It also suppressed Top2 and caused DNA double-strand breaks. It disrupted microtubule more potently than Top2. YCH337 induced reversible mitotic arrest at low concentrations but persistent DNA damage. YCH337 caused intrinsic and extrinsic apoptosis and decreased MCL-1, cIAP1 and XIAP proteins. In this aspect, YCH337 behaved differently from the combination of vincristine and etoposide. YCH337 inhibited proliferation of tumor cells with an averaged IC_50_ of 0.3 μM. It significantly suppressed the growth of HT-29 xenografts in nude mice too. Importantly, YCH337 nearly equally killed different-mechanism-mediated resistant tumor cells and corresponding parent cells. Together with the novelty of its chemical structure, YCH337 could serve as a promising lead for drug development and a prototype for a dual microtubule/Top2 targeting strategy for cancer therapy.

## INTRODUCTION

Both microtubule and topoisomerase II (Top2) are important anticancer targets and their respective inhibitors have been widely used for cancer therapy [1–4]. Microtubule consists of microtubulin. The dynamic equilibrium between polymerization and depolymerization of microtubulin precisely regulates the function of microtubule [3]. Interference with this balance by promoting polymerization or depolymerization disrupts microtubule and thus impairs the mitotic spindle assembly. Consequently, cells are arrested in M phase, causing proliferative inhibition or even cell killing [5]. Tubulin inhibitors can be roughly classified into microtubule stabilizers (*e.g*., taxanes) and destabilizers [*e.g*., *Vinca* alkaloids] [6]. Taxanes bind to the paclitaxel site and promote microtubule polymerization while *Vinca* alkaloids bind to the vinblastine site and accelerate depolymerization. Both have been extensively used in the clinic. There are other microtubule destabilizers such as combretastatin A4 (CA4) that bind to the colchicine site to depolymerize microtubule, which are undergoing clinical trials [7]. In contrast, Top2, a nuclear enzyme, is critical for resolving DNA entanglement and for segregating chromosomes in mitosis [8]. Top2 catalytically cleaves the DNA duplex and mediates the passage of its one segment through another. This process generates transient Top2-DNA covalent complexes (Top2cc) and DNA double-strand breaks (DSB) that are normally rapidly repaired. Stabilizing the Top2cc results in the accumulation of DSB, which activates DNA damage response, subsequently leads to G1, S and/or G2 arrest and induces apoptosis. This is the basic mechanism of action of Top2 poisons (*e.g*., podophyllotoxins and anthracyclines), the most important subclass of Top2 inhibitors in the clinic [9].

Microtubule inhibitors and Top2 inhibitors are frequently used in combination for cancer therapy. Many therapeutic regimens contain vincristine (VCR), vinblastine, paclitaxel or docetaxel in combination with doxorubicin (DX) or etoposide (VP-16) for the treatment of various hematological or solid tumors [4]. These combinations have been reported not only to produce synergistic therapeutic effects but also to reduce nonhematologic toxicities [10]. However, there are also findings that improper combinations, for example, colchicine plus VP-16, might elicit antagonistic effects or enhance toxicities [11, 12]. Therefore, how to choose correct drug combinations and take proper sequential approaches becomes an important issue, the resolution to which remains largely empirical at present. Another issue is tumor drug resistance [13]. The use of microtubule inhibitors and Top2 inhibitors frequently leads to multidrug resistance (MDR) that is mediated usually by overexpressed drug transporters [14, 15]. Importantly, these MDR tumor cells are usually cross-resistant to both of them, causing treatment failures even when used in combination [11, 16, 17]. In contrast, mutations of the drug binding sites on microtubule or Top2 might also result in resistance, generally, only to their respective inhibitors (*i.e*., individual drug resistance, IDR) by reducing the effective drug binding. In this case, tumor cells resistant to Top2 inhibitors could still be sensitive to microtubule inhibitors and *vice versa*. For this reason, their combination is likely to delay the emergence of drug resistance or even combat it to some degree.

However, few single agents have been reported as yet to concomitantly inhibit microtubule and Top2. Theoretically, such agents could cause similar therapeutic efficacy as the combination of the two classes of inhibitors but make the drug choice and administration simpler and easier. Moreover, these agents could also overcome IDR or even MDR as described above, and thus achieve more persistent therapeutic effects. Here we show such a strategy of dual targeting of microtubule and Top2 to achieve anticancer effects, as exemplified by a single compound YCH337. This novel microtubule/Top2 inhibitor was shown to inhibit both microtubule and Top2, result in microtubule depolymerization and DSB, subsequently lead to mitotic arrest and apoptosis and finally elicit tumor proliferation and growth inhibition *in vitro* and *in vivo*. YCH337 was also revealed to kill both MDR and IDR tumor cells and their respective parent cells to an equivalent degree. In addition, we found that YCH337 behaved differently from the combination of the microtubule inhibitor VCR and the Top2 inhibitor VP-16, particularly in inducing apoptosis.

## RESULTS

### YCH337 inhibits microtubule polymerization by binding to the colchicine site, leading to spindle assembly disruption

**Figure 1:**
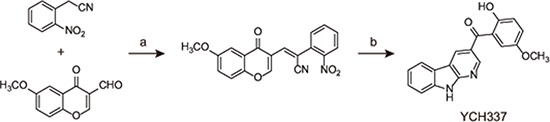
The chemical structure and synthesis of YCH337 Reagents and conditions used in the preparation of YCH337: **(a)** NaOAc, AcOAc, 90°C, 85%; **(b)** Fe, AcOH, reflux 2 h, 55%.

**Figure 2:**
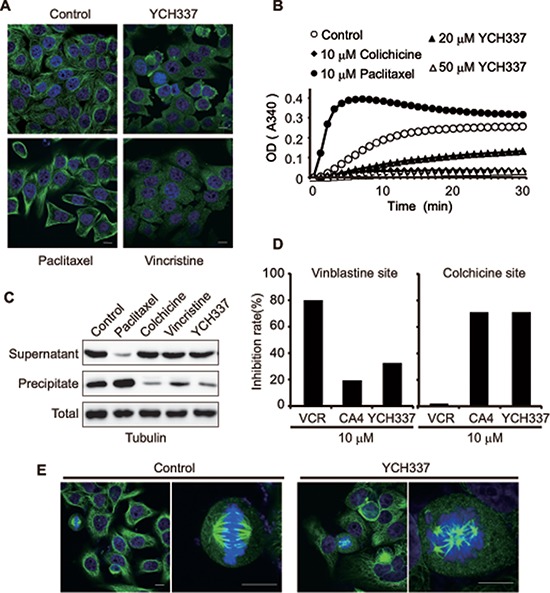
YCH337 inhibits microtubule polymerization by binding to the colchicine site and disrupts spindle assembly **(A)** HeLa cells were treated with YCH337, vincristine or paclitaxel at 0.5 μM for 1 h and then interphase microtubule was imaged by immunofluorescence microscopy. Scale bar: 10 μm. **(B)** YCH337 inhibited *in vitro* polymerization of microtubulin in a cell-free system. Colchicine and paclitaxel were used as references. **(C)** SK-OV-3 cells were treated with different agents at 0.2 μM for 24 h. Then, the polymerized fraction and the free fraction of tubulin were separated by ultracentrifugation and processed for Western blotting. **(D)** the binding site of YCH337 on tubulin was determined by the competitive binding assay. Vincristine and CA4 were used as references. **(E)** HeLa cells were treated with 1 μM YCH337 for 1 h, and then the mitotic spindle assembly was shown by immunofluorescence microscopy. Scale bar: 10 μm.

YCH337 {(2-hydroxy-5-methoxyphenyl) (9*H*-pyrido [2,3-*b*] indol-3-yl) methanone} [18] (Figure 1), a novel α-carboline derivative, was shown to cause cellular microtubule depolymerization (Figure 2A). It also inhibited tubulin polymerization in a concentration-dependent manner in a cell-free system (Figure 2B). Consistently, the treatment of HeLa cells with YCH337 increased the fraction of free tubulin (in supernatant) but decreased the fraction of polymerized tubulin (in precipitate), without changing the level of total tubulin (Figure 2C). Similar to the colchicine-site binder CA4, YCH337 competitively reduced the binding of colchicine to tubulin, but did not affect the binding of VCR to tubulin obviously (Figure 2D). Furthermore, the treatment with YCH337 resulted in multipolar spindle formation and chromosome misalignment in HeLa cells (Figure 2E). The data indicate that YCH337 inhibits cellular microtubule polymerization by binding to the colchicine site, thus disrupting mitotic spindle assembly.

### YCH337 inhibits Top2, which is weaker than it suppresses microtubule polymerization in cells

**Figure 3:**
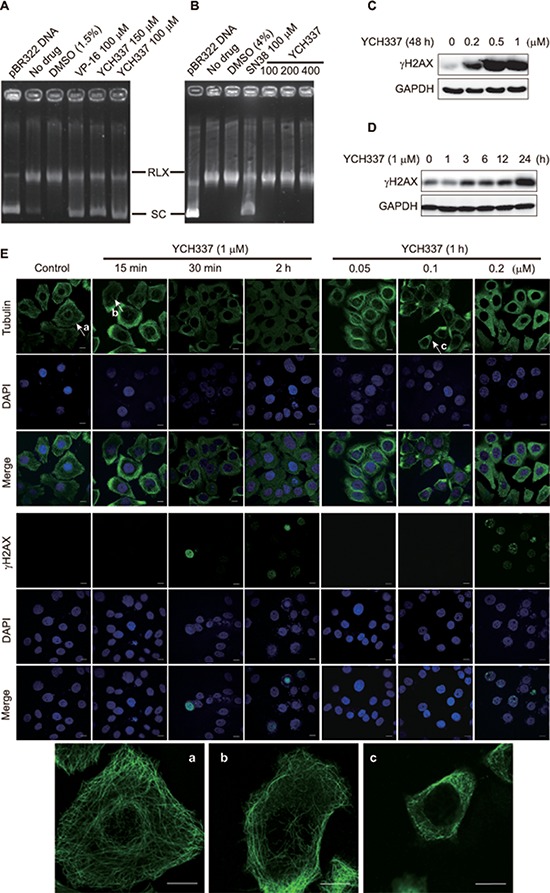
YCH337 inhibits Top2, which is weaker than it suppresses microtubule in cells **(A–B)** YCH337 inhibited DNA decatenation catalyzed by Top2 (A) rather than by Top1 (B). The electrophoresis assay was described in Materials and methods. Each reaction contained the same amount of Top2 (A) or Top1 (B) and DMSO except the control (pBR322 DNA). The Top2 inhibitor etoposide (VP-16) and the Top1 inhibitor SN38 were used as positive controls. RLX: the relaxed form of pBR322 DNA. SC: the supercoiled form of pBR322 DNA. **(C–D)** HeLa cells were treated with YCH337 at indicated concentrations for 48 h (C) or at 1 μM for the indicated time (D). Then the cells were lyzed and immunoblotted for γH2AX. **(E)** HeLa cells were treated with YCH337. Tubulin and γH2AX foci were then imaged by the immunofluorescence-based laser confocal microscopy. The magnified images of microtubule in the cells pointed to by the arrows (**a, b** and **c**) were presented at the bottom of the figure. Scale bar: 10 μm.

YCH337 apparently decreased pBR322 DNA relaxation mediated by Top2 (Figure 3A) rather than Top1 (Figure 3B) in cell-free systems. The treatment with YCH337 led to DSB in a concentration- (Figure 3C) or time- (Figure 3D) dependent manner, as revealed by the progressive increase in the levels of γH2AX [a well-documented molecular marker of DSB [19]] in HeLa cells. At single-cell levels, YCH337 also caused not only microtubule depolymerization but also the formation of γH2AX foci (Figure 3E). In contrast, the classical tubulin inhibitors only affected microtubule polymerization (Supplementary Figure S1). Noticeably, cellular microtubule depolymerization occurred as early as 15 min in the cells treated with 1 μM YCH337 (Figure 3E, a *versus* b) or at as low as 0.1 μM in the cells treated with YCH337 for 1 h (Figure 3E, a *versus* c). In those corresponding cells, however, the γH2AX foci began to form at 30 min or at 0.2 μM (Figure 3E). These data indicate that YCH337 is a dual tubulin/Top2 inhibitor, but its microtubule depolymerization is more potent than its Top2 inhibition.

### YCH337 induces reversible M arrest but irreversible DNA damage at a relatively low concentration

**Figure 4:**
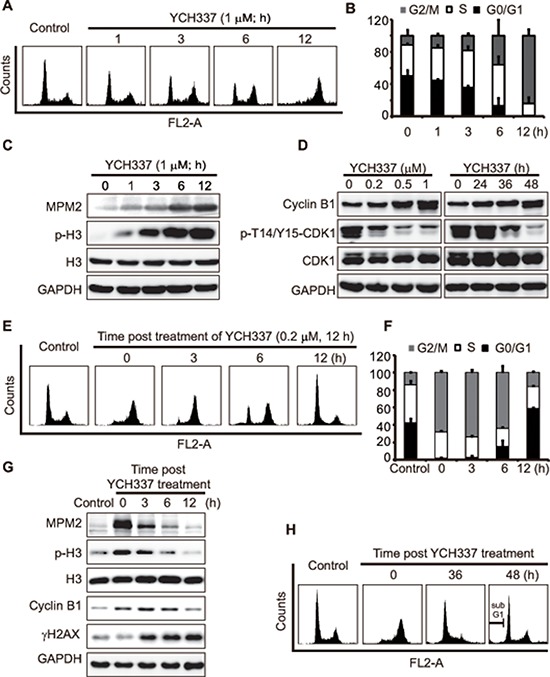
YCH337 induces reversible mitotic arrest but irreversible DNA damage **(A–C)** HeLa cells were treated with 1 μM YCH337 for the indicated time. Cell cycle was analyzed by flow cytometry (A). The statistical data of three independent experiments were shown in (B). The cells were treated with 1 μM YCH337 for the indicated time, and then the cells were lyzed and immunoblotted for mitotic molecular markers MPM2 and p-H3 (Ser10-phosphorylated histone 3) (C). **(D)** HeLa cells were treated with YCH337 at 0.5 μM for the indicated time or at indicated concentrations for 48 h. Then, the cells were lyzed and immunoblotted for CyclinB1 and phosphorylated CDK1 at Thr14/Tyr15 (p-T14/Y15-CDK1). **(E–F)** HeLa cells were treated with 0.2 μM YCH337 for 12 h. Then the medium was changed to remove YCH337 and the cells continued to be cultured in the drug-free medium for indicated time for flow cytometry analysis (E). The statistical data of three independent experiments were shown in (F). **(G–H)** HeLa cells were treated as in (E) and collected for Western blotting (G) or flow cytometry analysis (H).

Either tubulin or Top2 inhibitors can cause typical cell cycle arrest [8, 20]. However, the effect of dual tubulin/Top2 inhibitors on cell cycle progression is still unknown. Our results showed that YCH337 caused a typical G2/M arrest in HeLa cells in a time-dependent manner (Figure 4A and 4B). The levels of MPM-2 and Ser10-phosphorylated histone3 (p-H3), both of which are mitotic molecular markers [21], progressively increased in the YCH337-treated HeLa cells (Figure 4C). In addition, CDK1/cyclin B1 complexes are considered to be the switch to mitosis [22, 23]. YCH337 led to the decrease in the p-T14/Y15-CDK1 level but the increase in the cyclinB1 level (Figure 4D). The results indicate that YCH337 arrests the cells in M rather than G2 phase.

To examine the reversibility of the M arrest and DNA damage caused by YCH337, we treated HeLa cells with the compound, then withdrew it and continued to incubate the cells in drug-free medium. The 12-h incubation in drug-free medium after drug withdrawal appeared to almost completely reverse M arrest after the cells were pretreated with 0.2 μM YCH337 for 12 h (Figure 4E–4G). In contrast, the levels of γH2AX continued to increase (Figure 4G), indicating that DNA damage was persistent at this condition. Moreover, the prolonged drug-free incubation brought the cells into G1 arrest at the 36-h time point and subsequently caused apoptosis (marked by the sub-G1 peak) at the 48-h time point (Figure 4H). However, the 12-h pretreatments of HeLa cells with enhanced concentrations (0.5 μM or 1 μM) of YCH337 led to irreversible M arrest and subsequent cell death (Supplementary Figure S2). The data indicate that YCH337 induces reversible M arrest at a relatively low concentration; but even at as low as 0.2 μM, it elicits the effect of persistent Top2 inhibition and DSB.

### YCH337 induces apoptosis, different from that elicited by the combination of the tubulin inhibitor VCR and the Top2 inhibitor VP-16

**Figure 5:**
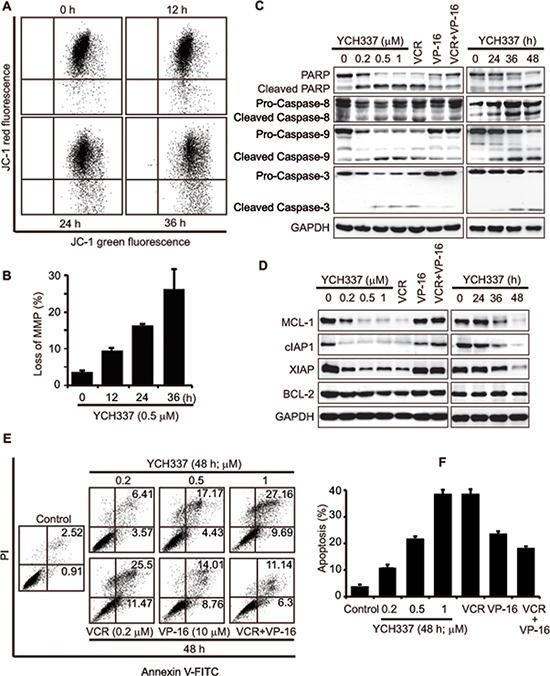
YCH337 induces apoptosis, different from that elicited by the combination of the tubulin inhibitor VCR and the Top2 inhibitor VP-16 **(A–B)** loss of mitochondrial membrane potential (MMP) in HeLa cells treated with 0.5 μM YCH337 for the indicated time was analyzed by flow cytometry. Representative images were presented in (A) and the data from three independent experiments were expressed as mean ± SD in (B). **(C–F)** HeLa cells were treated with different agents at the indicated concentrations for 48 h or with 0.5 μM YCH337 for the indicated time. VCR and VP-16 were used at 0.2 μM and 10 μM, respectively. Then cells were lyzed and immunoblotted for PARP and caspases (C) or anti-apoptotic proteins (D). Apoptosis was analyzed by the Annexin V-PI co-staining-based flow cytometry. Representative histograms were presented (E). The data from three independent experiments were expressed as mean ± SD (F).

Persistent mitotic arrest or DNA damage can trigger apoptosis [24–26] through intrinsic and/or extrinsic pathways [27]. The treatment with YCH337 caused loss of mitochondrial membrane potential (MMP) in HeLa cells in a time-dependent manner (Figure 5A and 5B). The treatment further resulted in the activation of caspase-8, caspase-9 and caspase-3 and the cleavage of PARP in concentration- and time-dependent manners (Figure 5C). The BCL-2 family proteins such as BCL-2 and MCL-1 regulate both intrinsic and extrinsic apoptosis by changing the mitochondria outer membrane permeabilization [28–31]. Inhibitor of apoptosis (IAP) proteins including cIAP1 and XIAP are negative regulators of caspases [32, 33]. YCH337 apparently reduced the levels of MCL-1, cIAP1 and XIAP in both concentration- and time-dependent manners but only marginally affected BCL-2 (Figure 5D). Further analysis revealed that YCH337 induced prominent apoptosis in both concentration- (Figure 5E and 5F) and time- (Supplementary Figure S3A and 3B) dependent manners. These data indicate that YCH337 induces apoptosis *via* both intrinsic and extrinsic pathways.

In inducing apoptosis, noticeably, YCH337 was similar to VCR but different from VP-16 and especially the combination of VP-16 with VCR. Either VCR or VP-16 alone led to the activation of caspase-8, caspase-9 and caspase-3, the cleavage of PARP and apoptosis, just as YCH337 did (Figure 5C, 5E and 5F). However, only VCR could also reduce the levels of MCL-1, cIAP1 and XIAP in HeLa cells, but VP-16 could not (Figure 5D). Moreover, VP-16 when combined with VCR could decrease the latter's activity including caspase-3 activation, antiapoptotic protein reduction and apoptotic induction (Figure 5C–5F).

### YCH337 inhibits proliferation and growth of human tumor cells *in vitro* and *in vivo*

**Figure 6:**
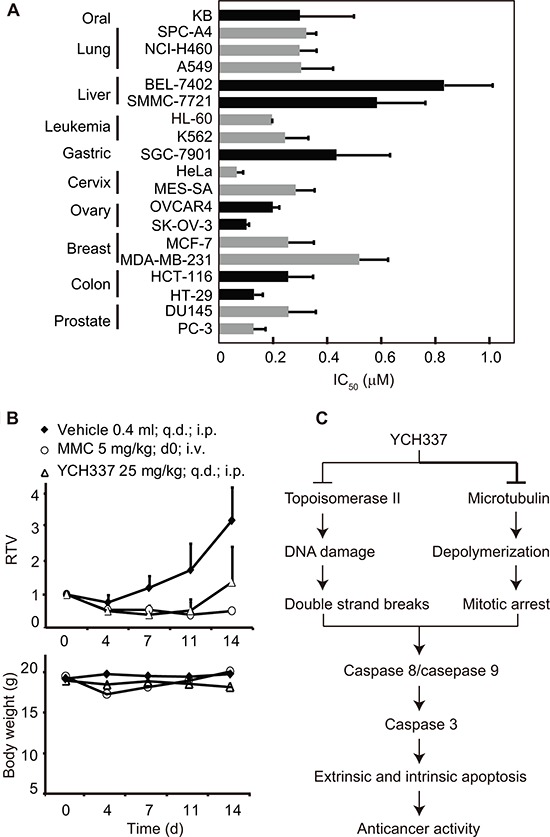
YCH337 inhibits proliferation and growth of human tumor cells *in vitro* and *in vivo* **(A)** YCH337 exerted potent antiproliferative activity on a panel of cancer cell lines. SRB assays (for solid tumor cells) or luminescent cell viability assays (for leukemia cells) were used to determine the 72-h IC_50_ values. Data from three independent experiments were expressed as mean ± SD. **(B)** YCH337 affected the growth of human colon cancer HT-29 xenografts in nude mice (upper) and the body weight of the tested animals (lower). Vehicle, 0.5% carboxymethylcellulose sodium; MMC, mitomycin C; RTV, relative tumor volume. There is statistically significant difference of RTV between the vehicle and YCH337 treatment groups beginning on day 7, *P* < 0.005. **(C)** a schematic presentation of possible mechanisms of action of YCH337.

We next examined the *in vitro* and *in vivo* activities of YCH337 against human tumor cells. YCH337 revealed potent proliferation inhibition in a panel of 19 tumor cell lines originating from 10 different tissues with an averaged IC_50_ value of 0.3 μM ranging from 0.06 μM (HeLa) to 0.83 μM (BEL-7402) (Figure 6A). Though the two liver cancer cell lines (BEL-7402 and SMMC-7721) seemed to have relatively low sensitivity, the profiling data appeared to show no apparent selectivity of the proliferation inhibition among the tested tumor cells. YCH337 is almost insoluble in water, which limits the possibility of its administration into animals by intravenous injection. We thus administrated YCH337 into animals by intraperitoneal injection to assess its *in vivo* anticancer activity. The data showed that YCH337 significantly suppressed the growth of human colon cancer HT-29 xenografts only with marginally reducing the body weight of the tested nude mice (Figure 6B). No animal death occurred in the experiment period. These data indicate that YCH337 possesses potent *in vitro* and *in vivo* anticancer activities with a broad anticancer spectrum.

### YCH337 kills both resistant and parental tumor cells to an equivalent degree

Finally, we determined the effect of YCH337 on drug-resistant cancer cells. KB/VCR and MES-SA/DX5 are MDR cancer cells lines established with the tubulin inhibitor VCR and the Top2 inhibitor DX, respectively. The two cell lines overexpress drug transporters such as P-gp and are highly resistant to VCR, DX and other various anticancer drugs [34, 35] (Table 1). However, YCH337 killed both of them, even a little more potently than it killed their respective parent cells, suggesting that the MDR mechanisms in these cells do not limit its *in vitro* activity.

**Table 1:** Effects of YCH337 on drug-resistant tumor cells

Agents	IC_50_ (μM)		RF	IC_50_ (μM)		RF	IC_50_ (μM)		RF
	KB	KB/VCR		MES-SA	MES-SA/DX5		HL60	HL60/MX2	
YCH337	0.43 ± 0.10	0.33 ± 0.07	0.8	0.28 ± 0.07	0.16 ± 0.04	0.6	0.21 ± 0.07	0.32 ± 0.01	1.5
VCR	0.01 ± 0.01	0.40 ± 0.17	40	–	–	–	–	–	
DX	–	–		0.09 ± 0.07	3.30 ± 0.44	37	–	–	
MX	–	–		–	–	–	0.06 ± 0.04	0.75 ± 0.23	12.5

NOTE: IC_50_ from three separate experiments were expressed as mean ± SD. Resistance factor was calculated as the ratio of the IC_50_ value of the drug-resistant cells to that of the corresponding parent cells.

Abbreviations: RF, resistance factor; VCR, vincristine, DX, doxorubicin; MX, mitoxantrone.

On the other hand, the HL60/MX2 cell line, established with the Top2 inhibitor MX, is resistant basically to Top2 inhibitors, due to its Top2 mutation rather than drug-transporter overexpression [36] (Table 1). Our data showed that both HL60/MX2 and its parent HL60 cell lines are highly sensitive to the dual tubulin/Top2 inhibitor YCH337 when compared to the Top2 inhibitor MX (Table 1). The result indicates that even when one drug target (*i.e*., Top2) is mutated, YCH337 still retains its possible cell killing through the other drug target (*i.e*., tubulin).

## DISCUSSION

Tubulin inhibitors and Top2 inhibitors are two classes of the most important antitumor drugs in the clinic [37, 38]. They are frequently used in combination due to their different mechanisms of action but faced with the issue of how to choose correct drug combinations and take proper sequential approaches. Moreover, almost all of them could lead to MDR and achieve only very limited therapeutic efficacy in MDR tumors. In addition, mutations in their respective targets could also confer drug resistance to them. Here we show a new potential anticancer strategy by dually targeting microtubule and Top2, as exemplified with the novel compound YCH337, achieving potent anticancer effects even in MDR cells or Top2-mutated cells.

Our data indicate that YCH337 leads to microtubule depolymerization by binding to the colchicine site and Top2 inhibition. Thus YCH337 disrupts spindle assembly and causes DSB damage. Consequently, mitotic arrest and subsequent apoptosis occur, which finally result in its proliferation inhibition in tumor cells and growth suppression in tumor xenografts (Figure 6C). Several characteristics of these activities are noticeable. (1) YCH337 targets both microtubule and Top2, but its microtubule depolymerization seems more potent than its Top2 inhibition. This is reflected in its microtubule depolymerization occurring earlier or at lower concentrations than DNA damage and its M arrest. (2) M arrest is reversible but DNA damage is persistent in the YCH337-treated cells, although both are irreversible at its relatively high concentrations (*i.e*., 0.5 μM or above). This result suggests the reversible binding of YCH337 to the colchicine site on one hand and its weaker but more lasting Top2 inhibition on the other, in considering no reported deficient repair for DSB in the tested HeLa cells. (3) YCH337 induces apoptosis through both intrinsic and extrinsic pathways. This might be potentiated by its reducing antiapoptotic proteins including MCL-1, cIAP1 and XIAP. However, the precise mechanisms still remain to be clarified. (4) YCH337 elicits non-selective proliferation inhibition in a panel of 19 tumor cell lines originated from 10 different tissues, suggesting its possible broad-spectrum antitumor activity. (5) YCH337 effectively kills drug resistant tumor cells and respective parent cells to an equivalent degree. Importantly, these drug resistant tumor cells were established with either tubulin inhibitors or Top2 inhibitors and overexpress drug transporters or carry mutated Top2 [35, 36, 39]. This feature of YCH337 is likely to open the door for the dual tubulin/Top2 targeting strategy for cancer therapy due to extensive clinical uses of tubulin inhibitors in combination with Top2 inhibitors and frequent emergence of drug resistance to them. Nevertheless, we have not yet known the effect of YCH337 in cells containing the mutated colchicine site because such cells are unavailable at present.

Although tubulin inhibitors and Top2 inhibitors are widely used in combination in the clinic and some combinations have been shown to mutually potentiate the activities in leukemia and solid tumor cells [40], VP-16 was reported to decrease the activation of caspase-3 and the induction of apoptotic cell death by colchicine (a tubulin inhibitor though not used as an anticancer drug in the clinic) in HEp-2 cells [11, 12]. In our study, similarly, when combined with the clinically-used tubulin inhibitor VCR, VP-16 was shown to antagonize the apoptosis-inducing activity of VCR, as evidenced by causing less caspase-3 activation, no antiapoptotic protein reduction and lower apoptotic induction. These data indicate that not all combinations of tubulin inhibitors with Top2 inhibitors could achieve synergistic therapeutic effects, further strengthening the importance of how to choose correct drug combinations in the clinic. However, YCH337, as a single agent dually targeting microtubule and Top2, shows prominent apoptotic induction without the above issue of VP-16 in combination with VCR or colchicine. From this perspective, a strategy of dual targeting of microtubule and Top2 to achieve anticancer effects might be feasible, though needing more examples and data to support.

The α-carboline derivative YCH337 has a novel chemical structure, bearing no structural analogy with either tubulin inhibitors or Top2 inhibitors currently used in the clinic [5, 9]. Neither have we found any reports on α-carboline derivatives inhibiting microtubule or Top2. Structurally, YCH337 contains two major moieties of 5-methoxyphenyl and α-carboline connected by a carbonyl group. The 5-methoxyphenyl moiety of YCH337 is similar to the methoxyphenyl moiety of the tubulin inhibitors MT7, MT119 and MT189, which is critical for their binding to the colchicine site and thus inhibiting tubulin polymerization [21, 41, 42]. On the other hand, the α-carboline moiety of YCH337 is similar to the β-carboline moiety of β-carbolines derived from the marine alkaloid manzamine A or arborescidine alkaloids, which is responsible for their Top2 inhibition [9, 43, 44]. Therefore, the two 5-methoxyphenyl and α-carboline moieties of YCH337 are likely associated with its microtubule depolymerization and Top2 inhibition, respectively. However, detailed structure-activity relationships remain to be established, which are critical for further demonstrating its mechanisms of action and improving its anticancer activity and physicochemical properties for drug development.

Together, our study presents a novel dual tubulin/Top2 inhibitor YCH337 with potent anticancer activity and drug-resistance-overcoming activity. It could act as a prototype for the dual tubulin/Top2 targeting strategy for cancer therapy. Such a strategy is likely to make the conventional combinations of tubulin inhibitors and Top2 inhibitors easier and simpler and to reduce the occurrence of drug resistance. Moreover, due to the novelty of its chemical structure, YCH337 could serve as a promising lead for future drug development.

## MATERIALS AND METHODS

### Drugs, chemicals and reagents

YCH337 was prepared according to our previous procedure [18] (Figure 1) with the purity over 98% determined by high performance liquid chromatography. Paclitaxel and CA4 were purchased from Sigma-Aldrich (St. Louis, MO). Colchicine was obtained from Sangon (Shanghai, China). VCR, DX and mitoxantrone (MX) were obtained from Melonepharma (Dalian, China). BODIPY FL-vinblastine was purchased from Invitrogen (Carlsbad, CA); mitomycin C (MMC) was purchased from Kyowa Hakko Kogyo Co. Ltd (Tokyo, Japan). All the reagents used *in vitro* were dissolved at 10 mM in 100% dimethyl sulfoxide (DMSO) as a stock solution and the aliquots were stored at −20°C. Prior to each experiment, all the chemicals were diluted to desired concentrations in normal saline immediately. YCH337 used *in vivo* was suspended in 0.5% carboxymethylcellulose sodium; MMC was dissolved in normal saline. Antibodies against histone H3 (sc-10809), phospho-histone H3 (Ser10) (sc-8656R), PARP (sc-7150), MCL-1 (sc-819), BCL-2 (sc-492), caspase-8 (sc-7890) and γH2AX (sc-101696) were from Santa Cruz Biotechnology (Santa Cruz, CA), caspase-3 (#9662s), and caspase-9 (#9502s) from Cell Signaling Technology (Danvers, MA), cIAP1 (ab2399), XIAP (ab28151) and Tubulin (ab7291) from Abcam (Cambridge, UK), GAPDH (AG019) from Beyotime (Shanghai, China), and MPM-2 (05–368) from Merck Millipore (Billerica, MA). The Kinase-Glo Luminescent Kinase Assay kit (V6713) was purchased from Promega (Madison, WI). Tubulin protein (T238P) and tubulin polymerization HTS assay kits (BK004P) were obtained from Cytoskeleton (Denver, CO).

### Cell culture

Human cancer A549, HT-29, HCT-116, KB, HeLa, PC-3, DU145, HL60/MX2, MES-SA/DX5 and MES/SA cell lines were purchased from the American Type Culture Collection (ATCC; Manassas, VA). Human cancer SGC-7901, BEL-7402, SPC-A4, SMMC-7721, MDA-MB-231, K562 and HL60 cell lines were kept in the Shanghai institute of *Materia Medica* of the Chinese Academy of Sciences (Shanghai, China). MCF-7, OVCAR-4 and SK-OV-3 were obtained from the Japanese Foundation of Cancer Research (Tokyo, Japan). The VCR-selected resistant KB/VCR cell line was purchased from the SunYat-Sen University of Medical Sciences (Guangzhou, China). The cell lines were cultured according to the suppliers’ instructions.

### Proliferation inhibition assays

The IC_50_ values of different agents in adherent and suspension cells were measured by the sulforhodamine B (SRB; Sigma, MO) assay and luminescent cell viability assay (Promega, Madison, WI) respectively, as reported previously [42, 45]. Cells were seeded into 96-well plates, cultured overnight and treated with gradient concentrations of the tested agents for 72 h. Optic density for both assays was read with an EnVision Multilabel Reader (PerkinElmer, Waltham, MA). The averaged IC_50_ values were determined with the Logit method from three independent experiments.

### Western blotting

Western blotting was performed as previously described [21].

### Immunofluorescence-based laser confocal microscopy

Cells were seeded on glass cover slips and cultured for 24 h, and then treated with YCH337 for the indicated time. Next, the cells were fixed with 4% paraformaldehyde for 30 min, permeabilized with 0.2% TritonX-100 at room temperature for 5 min. After that, the cells were blocked with 3% bovine serum albumin (BSA) at 4°C overnight, incubated with the primary antibody for 1 h and stained with fluorescence-conjugated secondary antibody at room temperature for 30 min. Finally, the cells were counter-stained with DAPI and imaged with an Olympus confocal microscope (Olympus, Tokyo, Japan).

### *In vitro* tubulin polymerization assays

*In vitro* tubulin polymerization was assessed by the turbidity assay as previously described [46].

### Cellular tubulin polymerization assays

Cells were treated with different agents and harvested in the lysis buffer (100 mM PIPES pH 6.9, 1 mM EGTA, 1 mM MgCl_2_, 30% glycerol, 5% DMSO, 1% NP-40, 5 mM GTP and protease inhibitors). The polymerized tubulin fraction (precipitate) and the soluble tubulin fraction (supernatant) were separated and determined as previously described [21].

### Tubulin-site competitive binding assays

Tubulin (3 μM) was incubated with the tested agents at 37°C for 1 h, and then colchicine or BODIPY FL-vinblastine (final concentration: 3 μM) was added. After 30-min incubation at 37°C, the fluorescence was determined with a PerkinElmer fluorometer (PerkinElmer, Waltham, MA) [21, 42, 47].

### Cell cycle assays

Cells treated with YCH337 for the indicated time were collected and washed with PBS, fixed with pre-cooled 70% ethanol at 4°C. Staining went along in PBS containing 40 μg/ml RNase A and 10 μg/ml propidium iodide in the dark for 15 min. Cells (at least 1×10^4^ cells per sample) were collected with FACS Calibur (BD Biosciences, Franklin Lakes, NJ).

### Annexin V-FITC apoptosis detection

Cells treated with YCH337 for the indicated time were collected and washed with PBS. Then, cells were co-stained by using the AnnexinV-PI apoptosis detection kit (Keygen, Nanjing, China). Fluorescence of the cells was determined immediately by flow cytometry (BD Biosciences, Franklin Lakes, NJ).

### Mitochondrial membrane potential (MMP) detection

Cells treated with YCH337 for the indicated time were collected and washed with PBS. Then, the cells were stained by using a JC-1 kit (Keygen, Nanjing, China). MMP was analyzed with a FACS Calibur (BD Biosciences, Franklin Lakes, NJ).

### Top1 or Top2-mediated supercoiled pBR322 relaxation

DNA relaxation assays were conducted as described previously [48]. For Top1, reaction buffer contained 0.5 μg supercoiled pBR322 DNA (Takara, Japan), 1 unit of Top1 (Takara, Japan), 2 μl 10×DNA Top1 buffer (350 mM Tris-HCl, pH8.0; 720 mM KCl; 50 mM MgCl_2_; 50 mM DTT; 50 mM spermidine), 2 μl 0.1% BSA and sterilized distilled water up to 20 μl. For Top2, reaction buffer contained 0.25 μg supercoiled pBR322 DNA, 2 units of Top2 (TopoGEN, Port Orange, FL), 4 μl 10×DNA Top2 buffer (mixed by buffer A and B in the Top2 assay kit, TopoGEN, Port Orange, FL) and sterilized distilled water up to 20 μl. Relaxation reaction was done at 37°C for 30 min and stopped by adding 5 μl stop buffer (TopoGEN, Port Orange, FL). DNA electrophoresis was carried out in a 1% agarose gel in TAE buffer at 100 V for 1 h. DNA bands were stained in 1 μg/ml of GelRed solution (BioTium, Hayward, CA) and photographed with a Gel Document System (GELDOCXR^+^, Biorad, Berkeley, CA).

### *In vivo* anticancer activity

Human colon cancer HT-29 xenografts in nude mice were used to evaluate the anticancer activity of YCH337. The model was established by the transplantation of 5×10^6^ HT-29 cells subcutaneously on the right armpit of each 4–5 weeks old female BALB/cA nude mouse. When the average tumor volume reached 100–200 mm^3^, 24 nude mice were selected based on the tumor volume and randomly assigned into vehicle, positive control and YCH337 groups. For consecutive 14 days, the animals in the YCH337 group were daily dosed intraperitoneally with YCH337 (25 mg/kg/d). The animals in the positive control group were dosed intravenously with MMC (5 mg/kg) on Day 0. The animals in the vehicle group were daily injected intraperitoneally with 0.5% carboxymethylcellulose sodium. During the treatment period, the implanted tumors were measured with a caliper twice a week. The maximum width (X) and length (Y) of the tumor were measured and the volume (V) was calculated using the formula: V = (X^2^Y)/2. Then relative tumor volume (RTV) was calculated as follows: RTV = V_t_/V_0_. V_0_ was the tumor volume at the beginning of the treatments, and V_t_ was the tumor volume after treatments. The animal body weight was also measured at the same time. The experiments abided by institutional ethical guidelines of the Animal Care and Use Committee (Shanghai Institute of *Materia Medica*, Chinese Academy of Sciences, China).

### Statistical analysis

All the data, if applicable, were expressed as mean ± SD. Comparison between two groups was performed with the Student's *t*-test. *P* < 0.05 was considered statistically significant.

## SUPPLEMENTARY FIGURES


