# Supplementary material for: Dual targeting of microtubule and topoisomerase II by α-carboline derivative YCH337 for tumor proliferation and growth inhibition
Source: Oncotarget. 2015 Mar 27;6(11):8960–73. doi: 10.18632/oncotarget.3264 (PMC4496195; doi:10.18632/oncotarget.3264)
Supplement: Supplementary file 1 [file oncotarget-06-8960-s001.pdf]

SUPPLEMENTARY FIGURES

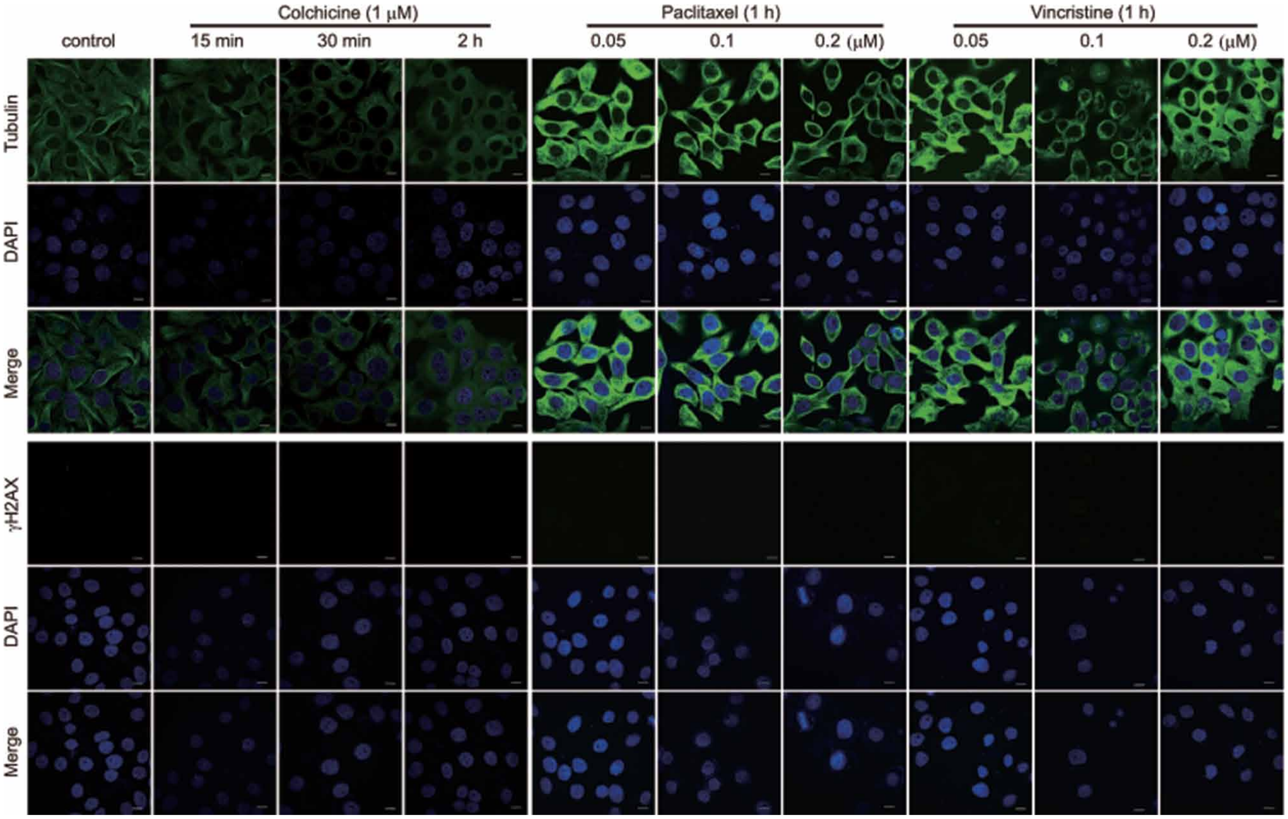

Supplementary Figure S1: Colchicine, vincristine and paclitaxel cannot induce the formation of γH2AX foci. HeLa cells were treated with colchicines, vincristine or paclitaxel. Tubulin and γH2AX were imaged by immunofluorescence-based laser confocal microscopy. Scale bar: 10 μm.

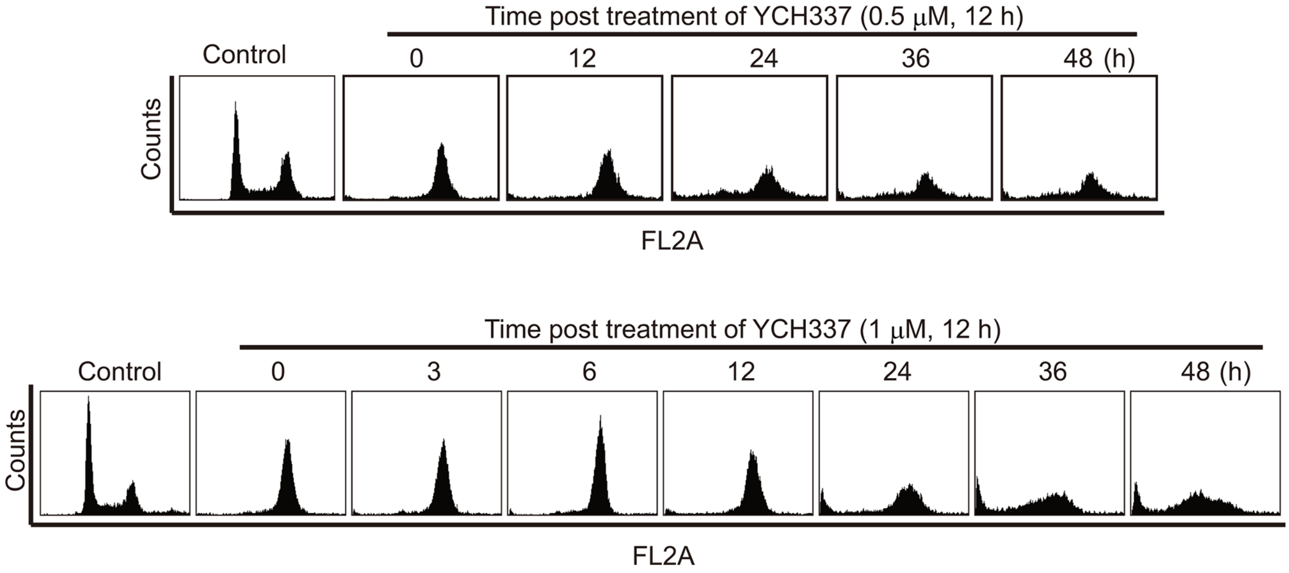

Supplementary Figure S2: Mitotic arrest caused by YCH337 is irreversible when the cells are treated at 0.5 μM or above for 12 h. HeLa cells were treated with 0.5 μM or 1 μM YCH337 for 12 h. Then the medium was changed to remove YCH337 and the cells continued to be incubated in the drug-free medium for indicated time for flow cytometry analyses.

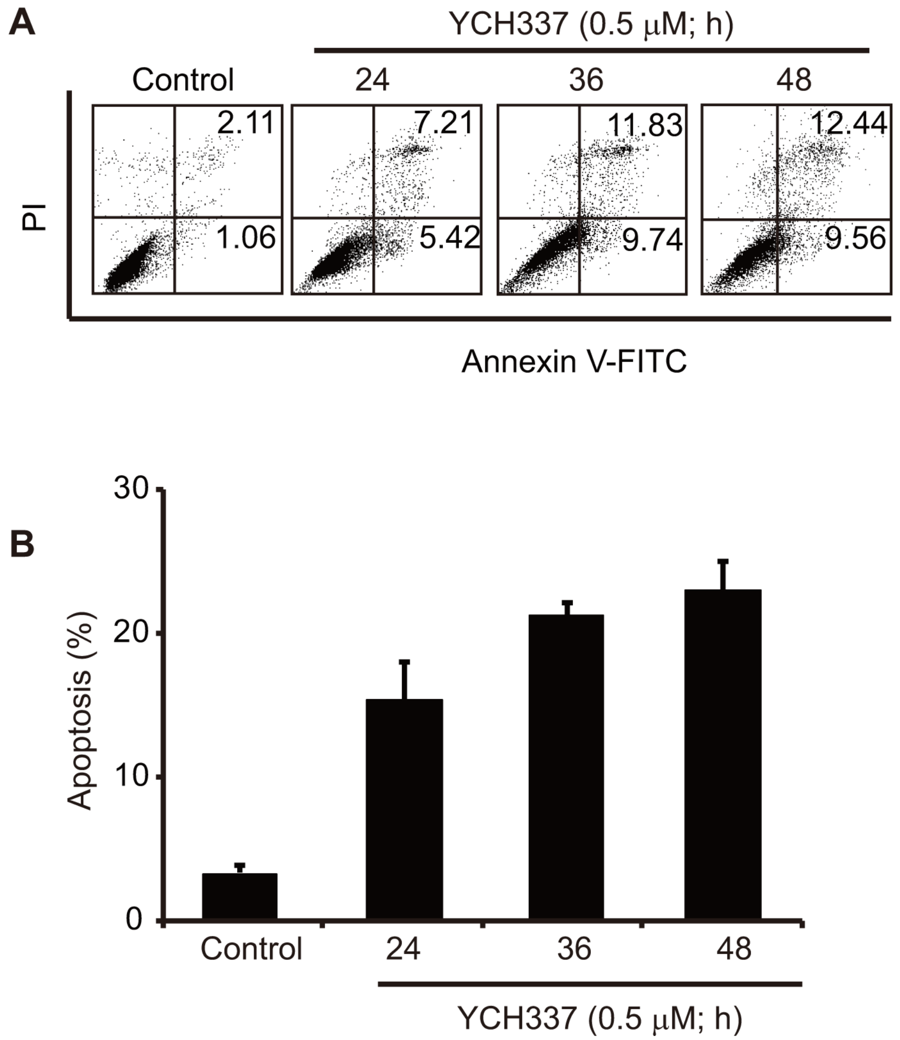

Supplementary Figure S3: YCH337 induces apoptosis. **(A–B)** HeLa cells were treated with YCH337 at 0.5 μM for the indicated time. Apoptosis was then analyzed by Annexin V-PI co-staining-based flow cytometry. Representative images were presented in (A) and the data from three independent experiments were expressed as mean ± SD in (B).
